# Liquid-based genomic profiling in high-risk localized prostate cancer

**DOI:** 10.3389/fruro.2026.1789586

**Published:** 2026-04-10

**Authors:** Fabiana Bettoni, Elisângela Monteiro Coser, Ernande Xavier dos Santos, Amanda Rafaela Alves Canteli, Romualdo Morandi Filho, Vandeclécio Lira, Livia Loureiro, Marcia Dellamano, Raul Torrieri, Maria José Ferreira Alves, Leonardo Cardili, David Queiroz Borges Muniz, Éder Nisi Ilário, Roger Chammas, William Carlos Nahas, Diogo Assed Bastos, Anamaria Aranha Camargo

**Affiliations:** 1 Hospital Sírio-Libanês, São Paulo, Brazil; 2 Illumina, Inc., San Diego, CA, United States; 3 Instituto do Câncer do Estado de São Paulo, São Paulo, Brazil

**Keywords:** biomarker, circulating tumor DNA, liquid biopsy, molecular profiling, prostate cancer

## Abstract

**Background:**

Tumor genomic profiling using liquid biopsies offers a minimally invasive alternative to tissue biopsy-based approach, with advantages in accessibility, tumor heterogeneity representation, and repeatability. While established in metastatic prostate cancer, its feasibility in localized disease remains unclear due to low ctDNA levels.

**Methods:**

We evaluated the feasibility and performance of tissue and liquid-based genomic profiling in patients with high-risk localized prostate cancer enrolled in a phase 2 neoadjuvant trial. Genomic DNA from formalin-fixed paraffin-embedded (FFPE) tissue and cell-free DNA from plasma were analyzed using Illumina TruSight Oncology 500 panels and DRAGEN™ pipelines, with in-house filtering of artifacts and germline variants.

**Results:**

Of 22 FFPE tissue biopsies, only 54.5% (12/22) yielded usable data, with failures due to low DNA quality or quantity. All 27 plasma samples (100%) were successfully sequenced, despite low ctDNA levels. Both approaches identified an average of 3.5 genomic variants per sample, including alterations in *SPOP*, *ATRX*, *ATM*, and *ARID1B*. Liquid-based genomic profiling achieved superior coverage and depth, enabling sensitive detection of low-frequency mutations. Concordance analysis was limited by the small number of matched samples (*n* = 4).

**Conclusions:**

Liquid-based genomic profiling is feasible and achieved high sequencing success rates in high-risk localized prostate cancer. Although concordance analysis was limited, plasma-based profiling showed robust sequencing performance and detected biologically relevant alterations. These findings support liquid biopsy as a complementary approach for molecular characterization, particularly when tissue samples are limited or of suboptimal quality. Larger studies are required to establish concordance and clinical utility.

## 1 Introduction

Circulating tumor DNA (ctDNA) genomic profiling using liquid biopsy represents an important tool for cancer management, with the potential to provide valuable prognostic and predictive information to guide therapeutic decisions and monitor disease progression and drug resistance (1). The minimally invasive nature of liquid biopsy is a significant advantage, as it permits repeated testing over the tissue-based approach and is suitable for patients who are unable to undergo biopsy due to tumor location or physical conditions. Additionally, liquid biopsies are more representative of spatial tumor heterogeneity than tissue biopsies, which may provide only an incomplete tumor genomic profile corresponding to the portion of the tumor sampled (2). Tissue-based genomic profiling is also associated with some notable limitations that the use of liquid biopsies can overcome; limited amounts of tissue obtained may result in insufficient DNA for sequencing; formalin-fixation and paraffin-embedding used for tissue preservation can damage the DNA and result in sequencing artifacts; low tumor cell content in the tissue biopsy may reduce test sensitivity.

Prostate cancer is the second most common type of cancer in men worldwide (3). It is a heterogenous disease, with a wide range of clinical manifestations that range from asymptomatic localized tumors to aggressive malignancies, which necessitates careful screening and risk assessment to avoid overtreatment and disease progression (1, 4). In addition to tissue availability, tumor content, and sampling site, the inherent heterogeneity of the disease can impact both the success rate of tissue-based tumor genomic profiling and the clinical interpretability of results. The technical success rate for genomic profiling using tissue biopsies in localized prostate cancer has been suboptimal at approximately 85%, with most failures due to inadequate tissue (5).

The use of liquid biopsy for tumor genomic profiling in advanced/metastatic prostate cancer is well documented; qualitative analysis of ctDNA can reveal mutational landscape of tumors, whereas quantitative analysis can provide information about tumor burden and disease progression, as ctDNA concentration has been shown to positively correlate with disease stages (1, 6). To date, limited evidence is available on the performance of liquid-based genomic profiling in localized prostate cancer, where ctDNA levels are expected to be low, however, regarding the use of liquid-based tumor genomic profiling in localized prostate cancer in which ctDNA concentration is generally low. Thus, this study was conducted to demonstrate the technical feasibility and robustness of plasma-based genomic profiling in high-risk localized prostate cancer.

## 2 Materials and methods

### 2.1 Clinical samples

Tissue and blood samples were obtained from 22 and 27 patients, respectively, who were diagnosed with high-risk localized prostate cancer. High-risk localized prostate cancer was defined as histologically confirmed stage 3 prostate cancer, and/or Gleason score of ≥ 8, and/or prostate-specific antigen level of ≥ 20 ng/mL. These patients had been recruited to a randomized phase 2 clinical trial to investigate the safety and efficacy of a neoadjuvant protocol based on androgen deprivation therapy combined with Abiraterone and Apalutamide at Instituto do Câncer do Estado de São Paulo (ICESP). Tissue and blood samples were collected before and after neoadjuvance therapy.

### 2.2 Mutation profiling

Formalin-fixed paraffin-embedded (FFPE) tumor tissues obtained by transrectal ultrasound-guided biopsies were macrodissected to enrich for at least 60% tumor purity. Tissue samples were collected at external services and subsequently sent to ICESP for specialized review. Genomic DNA was extracted using the GeneRead DNA FFPE kit (Qiagen, Germantown, MD) according to the manufacturer’s instructions. DNA integrity was assessed using the Illumina FFPE QC Kit and as previously described (7). DNA libraries were constructed using 10–30 ng of the extracted DNA, with TruSight Oncology 500 v2 library prep kit (Illumina, San Diego, CA). Paired-end 100 bp sequencing was performed on the NextSeq™ 500 platform (Illumina).

Blood samples were collected into EDTA tubes and processed within 2 hours of collection to obtain plasma samples. Briefly, blood samples were centrifuged at 800g for 10 min at 4 °C. Supernatants were collected and further cleared by centrifugation at 11, 000g for 10 min at 4 °C and immediately stored at ─80 °C. Cell-free total nucleic acid (TNA) was isolated using the MagMAX Cell-Free Total Nucleic Acid Isolation Kit (Thermo Fisher Scientific, Waltham, MA). Size-based quantification was performed on the Tape Station system using Cell-free DNA Screen Tape Analysis (Agilent Technologies, La Jolla, CA). DNA libraries were constructed using 10–30 ng of the extracted DNA, with TruSight Oncology 500 ctDNA v2 library prep kit (Illumina). Paired-end 150 bp sequencing was performed on the NovaSeq™ 6000 platform (Illumina).

Illumina DRAGEN™ version 2.6 was used to analyze data from both tissue and liquid biopsy-based sequencing. Additional analyses were performed using an in-house pipeline to filter (i) variants that were below the limit of detection of TruSight Oncology 500 v2 and TruSight Oncology 500 ctDNA v2; (ii) rare germline variants present in ABraOM database (8); (iii) sequencing artifacts presented in more than two samples and that were not described as a hotspot mutation in COSMIC database (V101, November 2024).

## 3 Results

### 3.1 Patient characteristics and specimens

Twenty-two FFPE tissue biopsy samples and 27 plasma samples from patients with high-risk localized prostate cancer were used for the study. The average age of the patients was 65 years, and most of them had tumors characterized as T3a, N0, and with a Gleason score >7 (**Table 1**). Of these samples, only four were matched between tissue and plasma samples.

**Table 1:** Clinicopathological characteristics of high-risk localized prostate cancer patients.

Clinicopathological characteristics		Tissue (*n*)	Plasma (*n*)
Tumor	T2	5	6
	T3a	12	13
	T3b	5	8
Lymph nodes	N0	22	25
	N1	0	2
Gleason score	6	1	2
	7	7	6
	8	7	8
	9	7	8
	10	0	3

### 3.2 Tissue biopsy

Of the 22 tissue biopsy samples received, 45.5% (10 out of 22) were not adequate for DNA sequencing due to several reasons: insufficient DNA quantity (*n* = 1), low DNA quality (*n* = 5), or low-quality sequencing results (*n* = 4). In the remaining 12 tissue biopsy samples that were successfully sequenced, we generated an average of 113 million passing-filter reads. Of these reads, approximately 98% aligned with the reference sequence of the human genome. The mean coverage of the target region was 201X, and on average, 76% of the target region achieved coverage greater than 100X.

**Figure 1:**
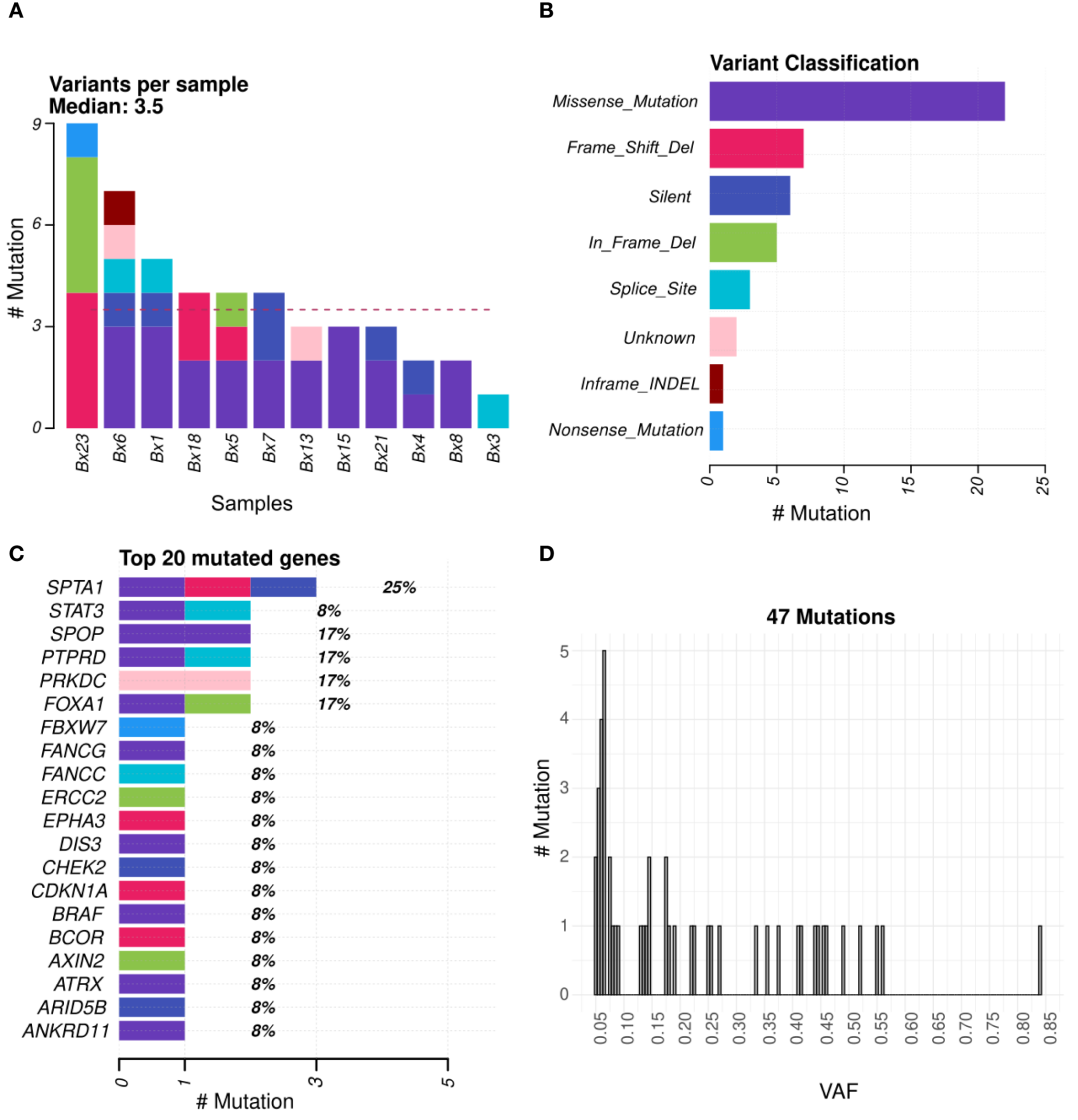
Molecular profiling of FFPE tumor tissues from patients with high-risk localized prostate cancer. **(A, B)** Number and distribution of somatic SNVs and Indels identified across tumor samples; **(C)** Top 20 most frequently altered genes identified in the cohort; **(D)** VAF distribution of the reported SNVs.

A total of 47 genomic variants were found in the 12 tissue biopsy samples with successful genomic profiling. At least one variant was detected in each sample, with an average of 3.5 variants per sample (**Figure 1A**). These genomic variants included missense and nonsense mutations, frameshift and in-frame deletions, silent mutations, and splice variants (**Figure 1B**). These alterations were detected in many genes, including key prostate cancer-associated genes (eg, *SPOP*, *ATRX*, and *CHECK2*) (**Figure 1C**). The variant allele frequency (VAF) was low as expected, due to the low tumor cell content in these samples (**Figure 1D**).

### 3.3 Liquid biopsy

Plasma genomic profiling was technically successful in all samples analyzed (27/27). However, concordance between plasma and matched tissue samples could only be evaluated in a limited subset of patients (n = 4). We generated an average of 932 million passing-filter reads. Of these reads, approximately 99.1% aligned to the reference sequence of the human genome. The mean coverage of the target region was 2, 381, and on average, 94.6% of the target region achieved coverage greater than 1, 000X.

**Figure 2:**
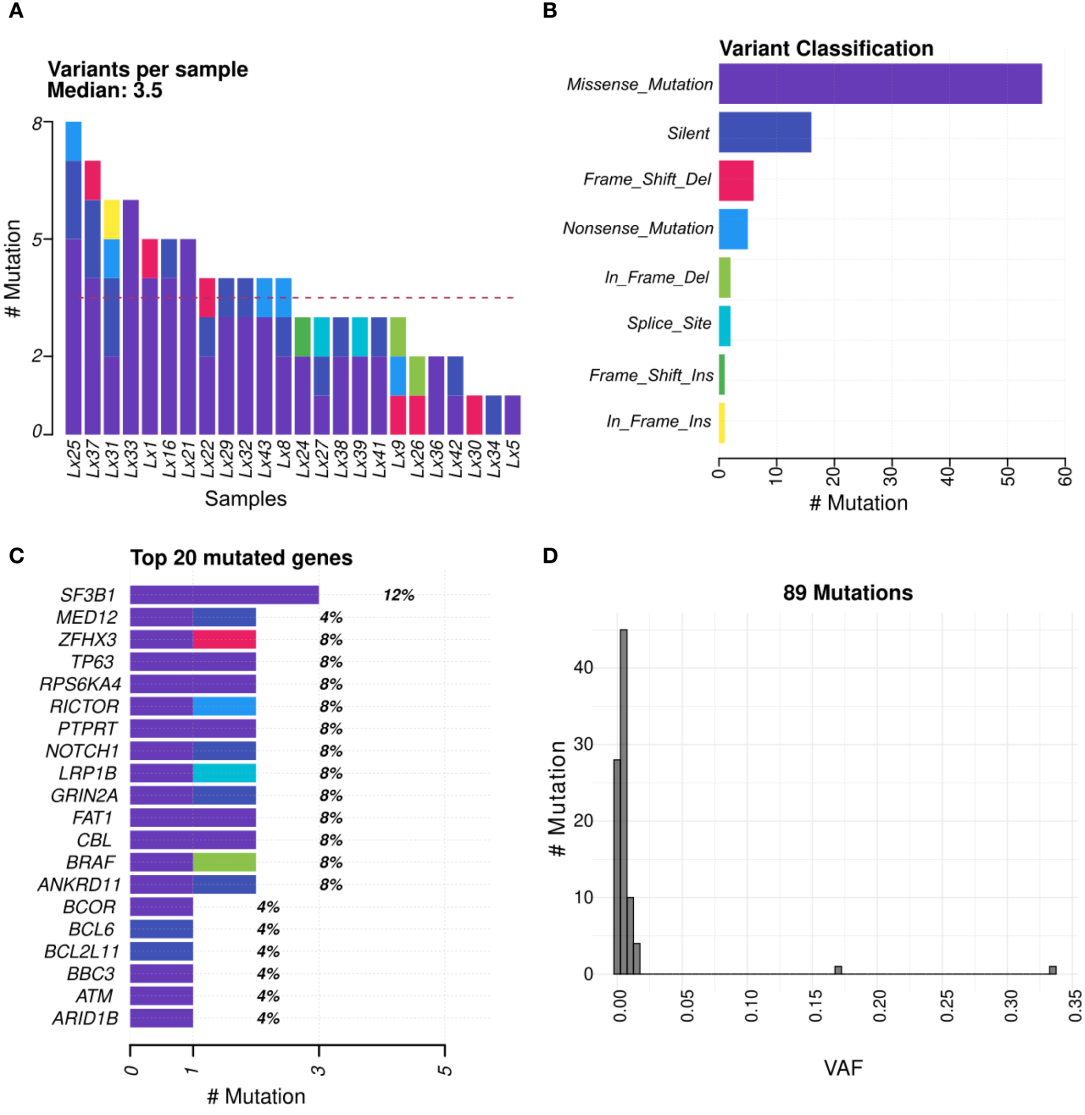
Molecular profiling of plasma samples from patients with high-risk localized prostate cancer. **(A, B)** Number and distribution of somatic SNVs and Indels identified across plasma samples; **(C)** Top 20 most frequently altered gene identified in the cohort; **(D)** VAF distribution of the reported SNVs.

Genomic variants found included missense and nonsense mutations, silent mutations, frameshift and in-frame insertions and deletions (indels), and splice variants (**Figure 2B**). They were detected in 89% of the sequenced samples (24 out of 27) with an average of 3.5 variants per sample (**Figure 2A**). Among the mutated genes were some of the notable prostate cancer-associated genes (eg, *ATM*, *NOTCH1*, and *ARID1B*) (**Figure 2C**). Low levels of ctDNA resulted in a very low VAF (**Figure 2D**).

## 4 Discussion

In this study, we assessed the feasibility and effectiveness of tumor genomic profiling using both tissue and liquid biopsies in patients with high-risk localized prostate cancer. Our findings underscore the technical challenges associated with FFPE tissue samples, while highlighting the potential of liquid biopsy to address some of these limitations.

Our results indicate a significant failure rate for tissue-based genomic profiling, with only 12 out of 22 tumor samples (54.5%) successfully sequenced. This low success rate was mainly attributed to DNA degradation caused by formalin fixation and long-term storage, along with insufficient tumor material. Prostate biopsies often contain limited tumor volume, being interspersed with benign glands and stroma that dilute tumor-specific signals and introduce sampling bias (9). These issues resulted in low DNA yield, low-complexity libraries, and poor sequencing quality metrics in nearly half of the cases. It is important to note that tissue samples were collected at external services and subsequently sent to ICESP for specialized review, therefore formalin quality, fixation and storage time were not controlled. Such challenges are consistent with previous reports on tissue-based genomic profiling in prostate cancer and other solid tumors, especially when using archival material or small core biopsies (5, 10).

Despite these limitations, the successfully sequenced tissue samples yielded high-quality sequencing data. With an average of 113 million reads per sample and a mean coverage of 201X, the assay provided reliable detection of genomic alterations, even at low VAFs. Notably, we identified 47 genomic variants across the 12 samples, encompassing a variety of mutation types. Importantly, we observed alterations in known prostate cancer driver genes, including *SPOP*, *ATRX*, and *CHEK2*, which are involved in chromatin remodeling, DNA repair, and tumor suppression pathways. These findings mirror those from The Cancer Genome Atlas (TCGA) and other large genomic studies of localized and advanced prostate cancer (11, 12), reinforcing the biological relevance of the detected variants.

In contrast, plasma genomic profiling was technically successful in all samples analyzed (27/27).Notably, 89% of plasma samples had detectable variants, exhibiting a mutational spectrum similar to that observed in the tissue samples as well as in previous mutational studies of localized and advanced prostate cancer (11, 12). The comparable number of genomic variants per sample (mean 3.5) between tissue and plasma samples is noteworthy, given the low ctDNA levels typically observed in localized disease.

In our study, the ctDNA detection rate was higher than what has been reported in previous studies. In a study involving a cohort of 112 patients with localized prostate cancer, neither ultra-low-pass whole-genome sequencing nor targeted resequencing detected ctDNA in preoperative plasma samples, including those from high-risk patients (13). Another study using patient-specific targeted sequencing found ctDNA in only 2 of 8 patients with clinically localized disease; the majority of localized cases showed undetectable levels of ctDNA, with preoperative detection associated with rapid disease progression to metastatic disease (14). Similarly, a larger cross-sectional study found that ctDNA detection rates were significantly lower in localized disease compared to metastatic prostate cancer (15).The higher detection rate observed in our study may reflect several factors. First, plasma samples were sequenced at ultra-deep coverage, with a mean target-region coverage of 2, 381X and greater than 1, 000X coverage across 94.6% of the target region, substantially increasing sensitivity for detecting low-frequency variants. Second, the use of a high-sensitivity targeted sequencing panel (TSO500 ctDNA v2), specifically designed for circulating tumor DNA analysis, enabled enhanced detection of clinically relevant alterations even at very low variant allele frequencies. Third, the application of optimized bioinformatic filtering pipelines, including artifact removal and germline and clonal hematopoiesis variants exclusion, likely improved signal-to-noise discrimination and variant calling accuracy. Finally, the high-risk clinical characteristics of the cohort may have also contributed to increased ctDNA shedding compared to lower-risk localized disease populations. Our results reinforce the growing body of evidence supporting the utility of liquid biopsy for genomic profiling in early-stage solid tumors (16, 17). Nevertheless, the low VAFs detected in both tissue and plasma highlight the need for highly sensitive bioinformatic pipelines and the notable impact of pre-analytical variables in ctDNA recovery.

A significant limitation of our study was the low number of matched tissue-plasma samples (*n* = 4), which prevented a comprehensive concordance analysis between the two approaches. However, the comparable mutation burden, mutation types, and presence of canonical driver alterations across both tissue and plasma suggest a degree of biological overlap. Prior studies have demonstrated varying levels of concordance between tissue and liquid-based genomic profiling, which are often influenced by tumor burden, spatial heterogeneity, and assay sensitivity (18, 19). In localized prostate cancer, tumor multifocality and the spatial separation of clones can lead to sampling bias in biopsy material, which may be partially mitigated by liquid biopsy that aggregates signals from multiple tumor foci.

It is important to emphasize, however, that the two approaches are not mutually exclusive but rather complementary. Tissue-based analysis remains essential for histopathological confirmation and assessments such as Gleason grading. It is also important to note that the American Society of Clinical Oncology currently do not recommend the routine use of molecular testing for localized prostate cancer, while recognizing the technical feasibility of such testing and the additional information it can provide (20). However, with the rapid development of novel therapies, liquid-based molecular profiling will surely become an attractive option to overcome pre-analytical issues associated with tissue-based testing and the heterogeneous nature of localized prostate cancer. Another important consideration is the potential value of relapse samples for longitudinal genomic monitoring. Previous studies have shown that certain mutations associated with therapeutic resistance can emerge under treatment pressure. Therefore, obtaining post-treatment samples, particularly at the time of disease progression, could offer critical insights into resistance mechanisms. While tissue re-biopsy may not always be feasible, especially in localized disease, liquid biopsy provides a minimally invasive alternative to track the evolution of the tumor’s molecular profile over time.

This study has several important limitations. First, as previously mentioned, the small sample size and limited number of matched tissue–plasma pairs precluded robust concordance analysis. Second, tissue samples were collected externally, and pre-analytical variables such as fixation conditions and storage duration were not controlled. Third, this study was not designed or powered to evaluate associations between ctDNA detection and clinical outcomes such as biochemical recurrence or progression-free survival. Fourth, direct comparisons between tissue and plasma sequencing metrics should be interpreted cautiously due to differences in sequencing platforms and depth, which were optimized for distinct analytical purposes and to reflect real-world clinical practice.

In summary, our findings demonstrate the technical feasibility and robustness of liquid-based genomic profiling in high-risk localized prostate cancer. While tissue biopsy remains the gold standard for histopathological diagnosis, liquid biopsy offers a non-invasive, repeatable, and highly informative tool that can complement existing diagnostic methods, especially when tissue samples are limited or of insufficient quality. Our findings support the integration of liquid biopsy into the diagnostic and monitoring processes for localized prostate cancer, potentially enabling more precise and individualized patient care. Further large-scale prospective studies are required to establish concordance, clinical validity, and clinical utility.

## Data Availability

The datasets presented in the study can be found in the European Genome-Phenome Archive (EGA; https://ega-archive.org/) under the accession number EGAS50000001712.
